# Paraneoplastic β-hCG secretion in a postmenopausal woman with sarcoma, endometrial carcinoma, and metastatic lung disease: a case report and review of the literature

**DOI:** 10.1186/s12905-026-04298-1

**Published:** 2026-01-26

**Authors:** Caixia Han, Chuanying Ding, Yilin Tan, Qianqian Zhao, Xiaofan Zhang, Weifeng Liang, Peihai Zhang

**Affiliations:** 1 Department of Gynecology, Cheeloo College of Medicine, Qilu Hospital (Qingdao), Shandong University, 758 Hefei Road, Qingdao, Shandong China; 2 Department of Pathology, Cheeloo College of Medicine, Qilu Hospital (Qingdao), Shandong University, 758 Hefei Road, Qingdao, Shandong China

**Keywords:** Human chorionic gonadotropin, Sarcoma, Diagnosis, Case report

## Abstract

**Background:**

Human chorionic gonadotropin (hCG) is a well-known diagnostic and prognostic marker for gestational trophoblastic neoplasm (GTN). Ectopic β-hCG production in sarcomas is rare, and its role in the pathogenesis and clinical outcomes in sarcoma has not been established. This case highlights the diagnostic challenge posed by markedly elevated serum β-hCG levels in a patient with concurrent sarcoma and endometrial carcinoma.

**Case presentation:**

A 60-year-old postmenopausal woman presented to the orthopedic department with complaints of progressive pain and discomfort in her left upper limb. Over the past nine months, she underwent two separate open reduction and internal fixation procedures for left humeral fractures following two separate incidents of minor trauma. Imaging studies revealed an osteolytic lesion in the humerus, a hypermetabolic lung nodule, and an intrauterine mass. Her serum β-hCG level was significantly elevated at 127,306 mIU/mL. Subsequent pathological analysis confirmed the diagnosis of undifferentiated sarcoma with lung involvement and concurrent endometrial cancer and ruled out the possibility of choriocarcinoma. Serum β-hCG monitoring showed a strong correlation between the variation and clinical course of the sarcoma, decreasing after tumor resection and increasing with disease progression and metastasis. A key analytical complexity was the consistent negative β-hCG staining on immunohistochemistry (IHC) across all tumor tissues (humerus, lung metastasis, and endometrium).

**Conclusion:**

A markedly elevated β-hCG level in a non-pregnant patient should not be automatically attributed to gynecological cancers. Undifferentiated sarcoma can be a rare source of massive ectopic β-hCG production, even with negative IHC staining.

**Supplementary Information:**

The online version contains supplementary material available at 10.1186/s12905-026-04298-1.

## Introduction

Human chorionic gonadotropin (hCG) is a well-established tumor marker for trophoblastic tumors and germ cell neoplasms [1, 2]. Despite sporadic reports, the biological basis, diagnostic relevance, and prognostic implications of β-hCG secretion in sarcomas remain poorly defined. Herein, we present a case of undifferentiated sarcoma in a female patient, in whom elevated serum β-hCG levels served as a paraneoplastic marker closely correlated with the clinical course, despite negative immunohistochemical staining in the tumor tissue.

## Case presentation

**Fig. 1:**
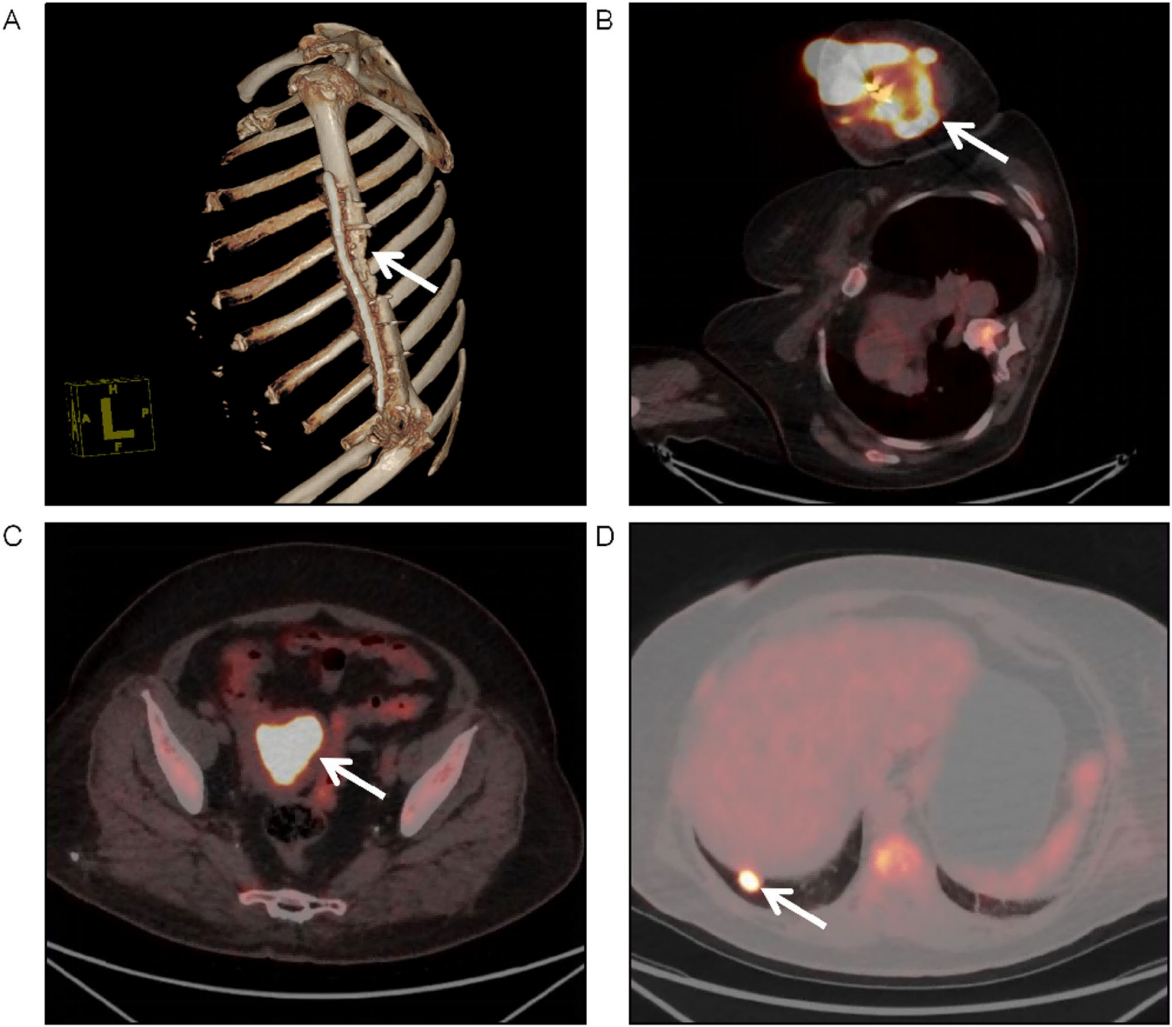
Radiographic evaluation before primary surgery. **A** Three-dimensional bone imaging of the left upper arm revealed a localized cortical defect in the left humerus, with an irregular bone contour in the humeral head. **B**-**D** Fused PET-CT images at presentation. **B** A large, hypermetabolic soft tissue mass (9.3 × 7.1 cm, SUVmax: 21.79) surrounding the left humeral shaft (arrow). **C** An intrauterine space-occupying lesion (4.5 × 3.4 cm, SUVmax: 14.31) with intense FDG uptake (arrow). **D** A hypermetabolic lung nodule (1.5 cm in diameter, SUVmax: 14.03) in the right lower lobe (arrow)

A 60-year-old postmenopausal woman presented to the orthopedic department with complaints of persistent pain and discomfort in her left upper limb. Nine months prior, she had undergone open reduction and internal fixation (ORIF) at a local hospital for a left humeral shaft fracture following a fall from a standing position. Postoperative recovery was uneventful. Five months prior, she had sustained a left humeral fracture after minor trauma. The patient underwent a second ORIF at the same institution. Following this revision surgery, the patient experienced progressive pain and functional impairment in the affected upper limb. Her medical history included a three-year history of hypertension. Her family history revealed that her father had died of esophageal cancer. The patient denied tobacco or alcohol consumption history. Physical examination revealed that the left upper arm and elbow exhibited significant swelling, with evident erythema and elevated local skin temperature. Radiography revealed localized bone defects in the left humerus of the patient. Initial workup, including a biopsy of the left upper arm lesion, suggested a poorly differentiated carcinoma. Concurrently, tumor marker tests revealed a significantly elevated serum β-hCG level of 127,306 mIU/mL. Subsequent Positron Emission Tomography-Computed Tomography (PET-CT) then revealed a hypermetabolic soft tissue lesion surrounding the left humeral shaft with significantly increased FDG uptake. Localized osteolytic destruction was also observed in the left humeral head, which exhibited a markedly enhanced FDG metabolic activity. The concurrent findings of intense FDG uptake in the intrauterine space-occupying lesion and hypermetabolic nodule in the right lower lobe of the lung raised concerns regarding metastatic disease or primary malignancy (Fig. 1). Disarticulation of the left shoulder joint accompanied by residual limb reconstruction was performed. Light microscopic examination of the resected sample revealed diffuse proliferation of markedly pleomorphic cells arranged in a haphazard pattern, predominantly composed of spindle-shaped cells. Immunohistochemical (IHC) staining was strongly positive for Vimentin, INI-1, BRG1, and Ki-67, and focally positive for MDM2. Negative expression was observed for SALL4, Desmin, CKpan, P63, CD31, CK8/18, S-100, SMA, SOX10, AFP, PLAP, GATA-3, MyoD1, CAM5.2, Melan-A, Muc-4, and HMB45. Fluorescence in situ hybridization (FISH) analysis demonstrated that MDM2 was not amplified. Based on the morphological and immunohistochemical findings, undifferentiated sarcoma was diagnosed. Histological evaluation of all nine axillary lymph nodes revealed no metastatic involvement, preserved nodal architecture, and no evidence of tumor infiltration.

**Fig. 2:**
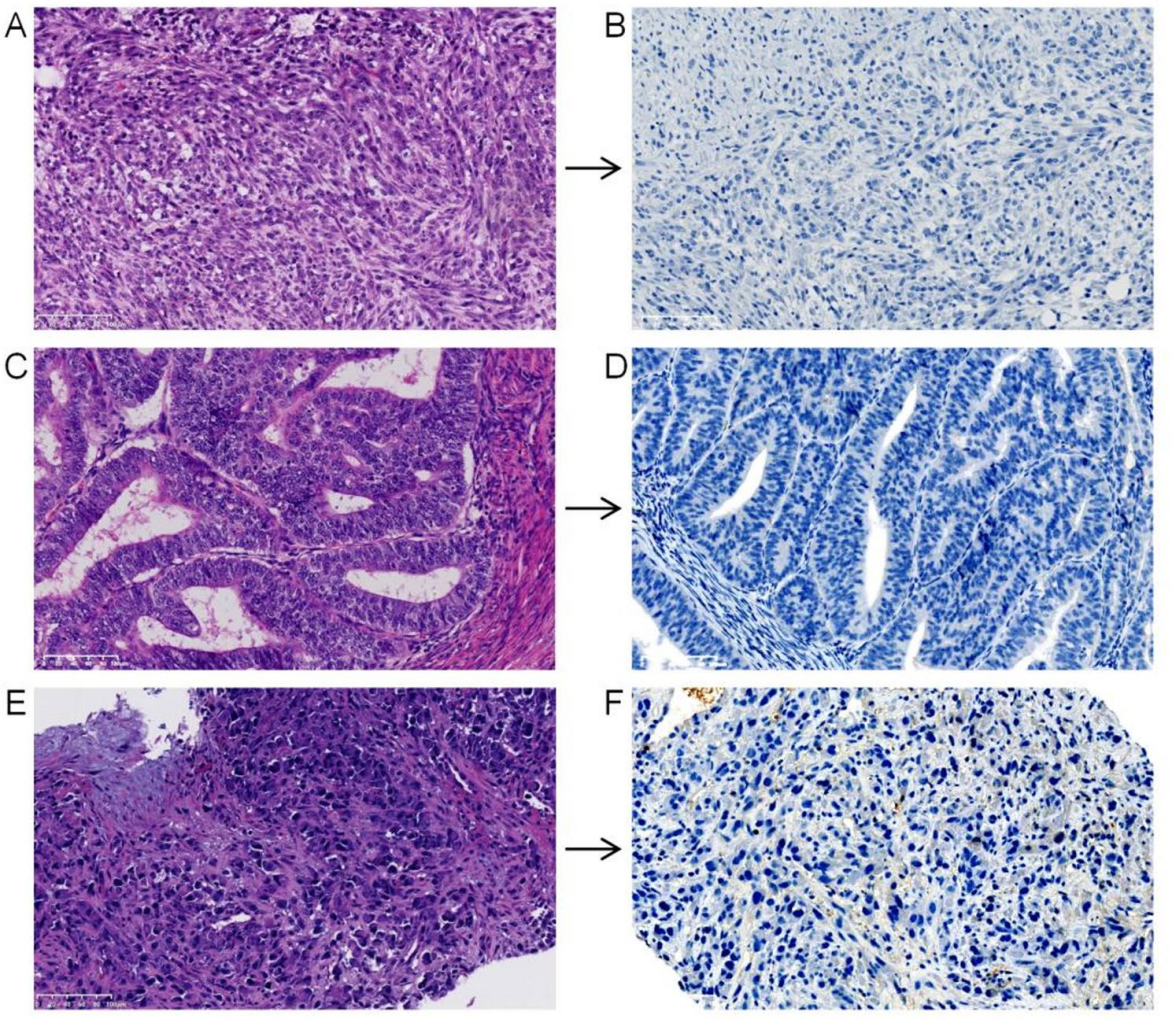
Histopathological and immunohistochemical analyzes of the tumors. Hematoxylin and eosin (H&E) staining (**A**, **C**, **E**) and β-hCG immunohistochemistry (**B**, **D**, **F**) of the primary left upper limb sarcoma (**A**, **B**), endometrial carcinoma (**C**, **D**), and lung metastasis (**E**, **F**). Scale bar: 100㎛

Due to the high level of β-hCG and intrauterine lesions, a gynecological consultation was requested for further evaluation. The patient had been menopausal for 10 years and had experienced irregular postmenopausal bleeding for the past two years. Her obstetric history included three full-term normal vaginal deliveries and two induced abortions. On pelvic examination, the cervix was closed; however, the uterus could not be adequately palpated because of the patient’s obesity. Endometrial biopsy revealed endometrial carcinoma (EC). Laparoscopic hysterectomy with bilateral salpingo-oophorectomy (TAH-BSO) was performed, and the final pathology report confirmed endometrioid adenocarcinoma (FIGO stage IB). A biopsy of the lung nodule confirmed metastatic undifferentiated sarcoma (IHC findings for key molecular markers are summarized in Figure S1). IHC staining (protocol details were shown in the Supplementary material) was negative for β-hCG in all examined lesions (Fig. 2). The final diagnosis was undifferentiated sarcoma with concurrent endometrial adenocarcinoma.

### Serial β-hCG monitoring and clinical course

**Fig. 3:**
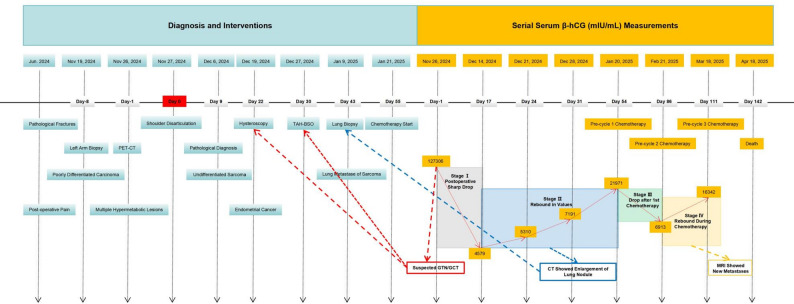
Timeline correlating dynamic β-hCG changes with disease trajectory. StageⅠ: Marked β-hCG elevation triggered GTN/GCT workup (hysteroscopy, TAH-BSO), subsequent pathology ruled out this diagnosis. Sharp β-hCG decline after shoulder disarticulation verified surgical efficacy and localized the β-hCG source to the left upper arm lesion. Stage Ⅱ: Asymptomatic β-hCG rise prompted chest CT, which showed enlarged lung nodules; subsequent biopsy confirmed sarcoma lung metastasis. Stage Ⅲ: β-hCG reduction after the first chemotherapy cycle indicated initial treatment response. Stage Ⅳ: Post-chemotherapy β-hCG rebound indicated disease progression and chemoresistance, as confirmed by MRI findings. Notes: “Day 0” corresponds to November 27, 2024 (date of shoulder disarticulation); negative “Day” values indicate events before Day 0, and positive values indicate events after Day 0

Following these diagnoses, serial monitoring of serum β-hCG levels revealed a dynamic correlation with the patient’s clinical course (Fig. 3). The serum β-hCG level dropped from 127,306 mIU/mL to 4,579 mIU/mL by post-disarticulation day 17. However, it rebounded to 5,310 mIU/mL on post-disarticulation day 24 and continued to rise, reaching 27,971 mIU/mL on post-disarticulation day 54. Chest computed tomography demonstrated significant enlargement of the right lower lung nodule, and biopsy confirmed metastatic sarcoma.

Owing to the diagnosis of advanced sarcoma with rapid progression, the multidisciplinary tumor board recommended systemic chemotherapy. The patient subsequently received three cycles of chemotherapy (epirubicin 75 mg/m² on Day 1 and ifosfamide 1.5 g/m² on Days 1–5, every 3 weeks), a first-line regimen endorsed by the NCCN guidelines. The treatment was associated with manageable grade 2 toxicities (nausea and neutropenia). Following the first cycle of chemotherapy, the β-hCG level dropped sharply to 6,913 mIU/mL. However, it rebounded to 16,342 mIU/mL after the second cycle. Imaging studies identified a metastatic lesion adjacent to the left clavicle and scapula. The patient ultimately succumbed to disease progression 30 days after the third cycle of chemotherapy.

## Discussion and conclusion

β-hCG is physiologically synthesized and secreted by placental syncytiotrophoblast cells during early gestation, where it plays a critical role in corpus luteum maintenance to support the pregnancy. Beyond physiological contexts, β-hCG is aberrantly secreted in multiple gynecological malignancies, including gestational trophoblastic disease, choriocarcinoma, and germ cell tumor (GCT) harboring neoplastic syncytiotrophoblastic elements. As a tumor-specific biomarker, its levels have significant prognostic relevance in these malignancies. Ectopic β-hCG expression has also been reported in multiple nongynecologic tumors, such as carcinomas of the lung, breast and prostate [3–6].

The production of β-hCG by sarcomas is an uncommon clinical entity that has rarely been documented. We searched PubMed/MEDLINE and Embase for English-language articles related to sarcoma and ectopic β-hCG secretion (using the specified keywords), excluding review articles. Only a few cases have been reported in the literature, which are summarized in Table 1 [7–25]. Of the 19 patients, 5 were male and 14 were female, with a median age of 41 years (range, 0.25-67). The predominant histological subtypes were osteosarcoma (6/19), leiomyosarcoma (5/19), and undifferentiated/pleomorphic sarcoma (3/19). Rare subtypes include synovial sarcoma, liposarcoma, cystosarcoma, and chondrosarcoma. β-hCG was detected via immunological assays of body fluids (serum, urine, or pleural fluid) and/or IHC of tumor tissue. The peak β-hCG levels exhibited an extreme range from 22.71 mIU/mL to 42,236 mIU/mL. Among the 17 cases with both available body fluids and IHC results, 15 (88%) were positive using both methods. Notably, the only two cases with negative IHC results had the lowest recorded serum β-hCG levels (22.71 and 48 mIU/mL), suggesting a potential threshold effect for tissue detection. Metastatic and/or recurrent disease was present at diagnosis or developed in 13 cases (68%), with the lungs being the most common site of metastasis. Among the 14 patients with available follow-up, 9 (64%) died of disease progression. The overall survival ranged from 3 weeks to 20 months, with a median survival of 5 months.

**Table 1:**
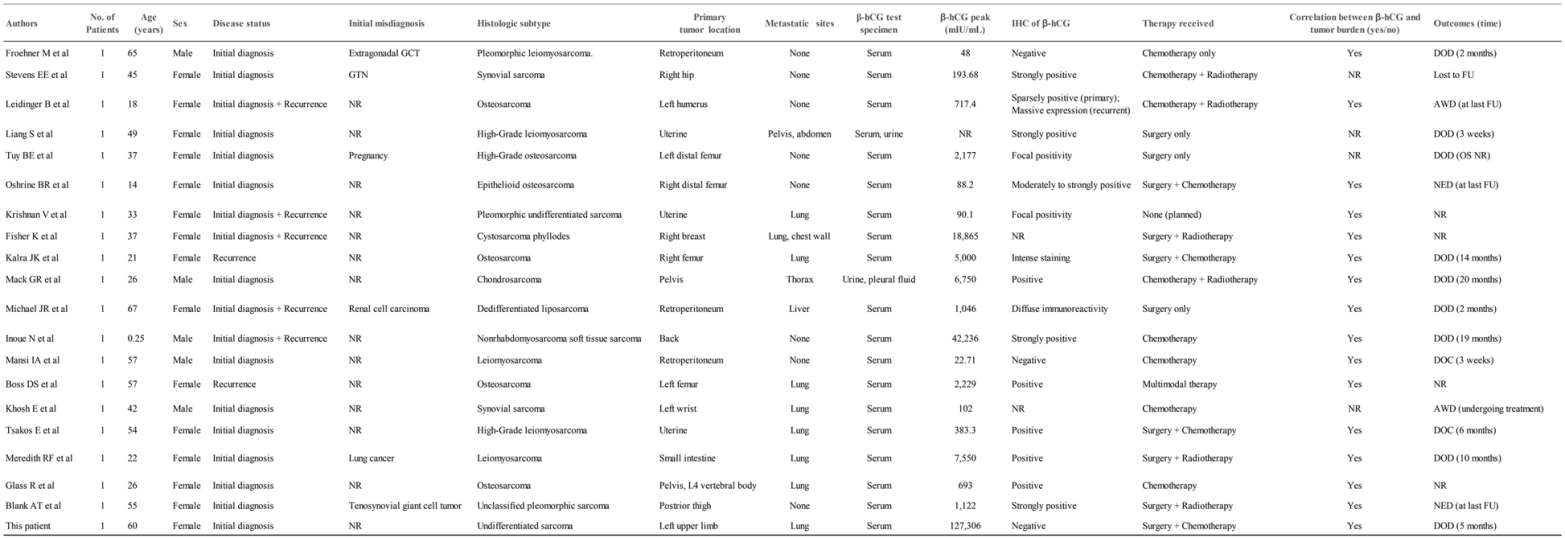
Clinical and pathological findings of reported sarcoma patients with ectopic β-hCG secretion

*Abbreviations: GTN* gestational trophoblastic neoplasm, *GCT* germ cell tumor, *AWD* alive with disease, *DOD* died of disease, *DOC* died of other causes, *NED* no evidence of disease, *OS* overall survival, *FU* follow-up, *NR* not reported

Compared with previously reported patients, the extraordinarily high β-hCG level made our case an extremely rare entity and raised concerns regarding potential analytical interference. Immunoassays are susceptible to various interfering factors, such as heterophile antibodies, the hook effect, and biotin, which can compromise the precision and reliability of the findings [26, 27]. To validate this result, we performed a dual-platform (Roche E801 and Beckman DxI 800) verification strategy, and detailed technical specifications are shown in Supplementary Table S1. The concordance between two distinct immunoassay platforms and assay kits confirmed that the measured β-hCG level is a true reflection of an active paraneoplastic secretion.

In this case, the combination of markedly elevated serum β-hCG levels and PET-CT findings indicating neoplastic lesions in both the uterine cavity and lungs presented a significant diagnostic challenge. Given the clinical presentation and high serum β-hCG level, gestational or non-gestational choriocarcinoma, EC with choriocarcinomatous differentiation, metastatic GCT, and carcinosarcoma were strongly suspected to be the cause. To specifically address the potential contribution of the EC components to the elevated β-hCG, we performed the following analyses. First, high-sensitivity IHC staining for β-hCG was conducted on all representative EC sections; however, no staining was detected in the tumor areas. The lack of both mononucleate trophoblastic cells and the characteristic dimorphic growth pattern, combined with the key negative IHC findings (β-hCG, SALL4, and GATA-3) in the tumor specimens, excluded the diagnosis of choriocarcinoma and any tumor with choriocarcinomatous differentiation. Carcinosarcoma was ruled out based on negative staining for a spectrum of epithelial markers (CKpan, CK8/18, CAM5.2, and P63) and the absence of typical epithelial morphology. Furthermore, GCT were unlikely because of negative staining for key markers, particularly SALL4, PLAP, and AFP. Critically, to definitively confirm the origin of β-hCG secretion, serum β-hCG levels were monitored after TAH-BSO. The serum β-hCG level increased from a preoperative value of 4,579 mIU/mL to 7,191 mIU/mL on the first postoperative day, and further rose to 21,971 mIU/mL by postoperative day 24. The observed perioperative β-hCG trend provided definitive evidence against the EC being the source of β-hCG secretion. Therefore, based on a comprehensive analysis of histomorphology, IHC, and molecular biology, the final diagnosis was confirmed as advanced undifferentiated sarcoma with pulmonary metastases, concurrent with EC.

The dynamic changes in serum β-hCG levels—a rapid postoperative drop followed by an increase with disease progression—strongly implicate sarcoma cells as the direct source of β-hCG secretion in this patient, despite negative β-hCG IHC staining in the sarcoma samples. The precise mechanisms underlying the discrepancies between IHC staining and biochemical assays for β-hCG remain unclear. Potential reasons for such discrepancies include low antigen concentration, prior chemotherapy, antibody sensitivity and specificity, mismatches between antibody target and hCG isoforms, and intratumoral heterogeneity [11, 15, 20, 22, 28, 29]. However, technical explanations for such discordance should first be considered, particularly pre-analytical variables related to tissue processing. The differential impact of formalin fixation is critical: prolonged ischemia before formalin fixation can induce autolysis and irreversible antigen degradation, while improper fixation duration itself can lead to under-fixation (resulting in antigen instability) or over-fixation (causing excessive protein cross-linking and epitope masking). In the present case, specimens were processed under standardized surgical pathology protocols, with minimal ischemic time and a calibrated formalin fixation period, followed by antigen-retrieval steps designed to reverse epitope masking. Therefore, while fixation variables represent an important general consideration, they are unlikely to be the primary cause of the observed discordance here. Furthermore, our patient presented with an extremely high serum β-hCG level (beyond 120,000 mIU/mL). In comparison, the two IHC-negative cases in Table 1 had levels below 50 mIU/mL. This marked difference indicates that a ‘threshold effect’ is an unlikely explanation here. Moreover, the IHC assay employed a high‑sensitivity monoclonal antibody targeting the core linear epitope (amino acids 21–165) of the β-subunit of hCG (Clone OTI12H5). Despite robust staining in positive controls, the tumor tissue remained entirely negative, effectively ruling out inadequate antibody sensitivity or specificity. After ruling out common technical artifacts by standardized protocols and rigorous quality control, we propose two biological explanations for the serum-tissue discordance in our patient. First, tumor-derived hCG exists as heterogeneous isoforms, including free β-subunit, hyperglycosylated hCG (hCG-H), and nicked variants, which may evade IHC detection via distinct mechanisms: hyperglycosylation (either on intact hCG-H or free β-subunit) occludes the conserved linear epitope, while proteolytic cleavage disrupts the epitope structure of nicked variants [30, 31]. The OTI12H5 antibody used in our study was raised against a recombinant fragment of the free β-subunit (aa 21–165), with specific recognition of unmodified free β-subunit, but it may not detect hyperglycosylated β-subunit isoforms or nicked variants with cleavage within the aa 21–165 epitope. Second, tumor cells may exhibit a ‘high-flux, low-pool’ secretory phenotype [32, 33]. This phenomenon leads to the immediate release of synthesized β-hCG. Consequently, hormones cannot accumulate intracellularly to levels detectable by standard IHC. Thus, negative IHC staining does not exclude paraneoplastic β‑hCG secretion by sarcomas, especially in the setting of extremely elevated serum levels.

Clinical evidence demonstrates that increased β-hCG expression, either systemically or locally, correlates with an adverse prognosis in diverse neoplasms [25, 26]. However, the clinical significance of ectopic β-hCG production in sarcomas remains unclear. Emerging evidence suggests that β-hCG secretion confirmed by IHC in osteosarcoma may serve as an indicator of chemotherapy resistance and a more aggressive phenotype [34]. However, the specific mechanism by which β-hCG is involved in tumor progression remains unclear. Iles RK identified structural homology between β-hCG and transforming growth factor beta (TGF-β) in adult epithelial cancer. Their findings suggest that ectopically expressed free β-hCG competitively binds to TGF-β receptors, potentially inhibiting TGF-β signaling and suppressing its pro-apoptotic effects [35]. As outlined in Table 1 (including our patient), regardless of the tumor’s osseous or soft tissue origin, the majority of these cases exhibited rapid disease progression. The high metastasis/recurrence rate (70%, 14/20) and shortened median overall survival (5 months) are indicative of aggressive biological behavior, implying that β-hCG secretion could be a potential prognostic indicator for unfavorable outcomes in sarcomas. In addition, 80% (16/20) showed a positive correlation between serum β-hCG levels and disease progression/therapeutic response, which increased with disease progression and decreased upon effective treatment. This suggests that serum β-hCG levels may serve as a biomarker for tumor burden and therapy evaluation. Therefore, based on the pattern observed in this and prior reported cases, we hypothesize that serial β-hCG monitoring could be of clinical value in sarcomas with aggressive phenotypes. To test this hypothesis in future studies, it would be reasonable to establish a baseline serum β-hCG level prior to treatment and perform serial measurements during therapy. In such a research context, a continued rise in β-hCG levels, even in the absence of new imaging findings, might alert clinicians to potential subclinical progression or emerging therapeutic resistance.

Collectively, markedly elevated β-hCG levels in non-pregnant patients should prompt consideration of sarcoma-derived paraneoplastic secretion, even when tumor IHC is negative. Recognizing this rare entity is essential to avoid misdiagnosis and treatment delays. Further studies are needed to elucidate the mechanisms underlying serum-tissue discordance observed in this case, as well as the utility and optimal application of β-hCG as a monitoring biomarker in sarcomas. Abbreviations: GTN, gestational trophoblastic neoplasm; GCT, germ cell tumor; AWD, alive with disease; DOD, died of disease; DOC, died of other causes; NED, no evidence of disease; OS, overall survival; FU, follow-up; NR, not reported.

## Supplementary Information

Supplementary Material 1.

Supplementary Material 2.

Supplementary Material 3.

## Data Availability

No datasets were generated or analysed during the current study.
