# Supplementary material for: Paraneoplastic β-hCG secretion in a postmenopausal woman with sarcoma, endometrial carcinoma, and metastatic lung disease: a case report and review of the literature
Source: BMC Womens Health. 2026 Jan 26;26:115. doi: 10.1186/s12905-026-04298-1 (PMC12918461; doi:10.1186/s12905-026-04298-1)
Supplement: Supplementary file 1 — Supplementary Material 1. [file 12905_2026_4298_MOESM1_ESM.docx]

#### Supplementary material

**Immunohistochemical Staining for β-hCG**

Tissues were processed routinely for microscopical examination: The studied tumor specimens were originally fixed in 10% neutral buffered formalin (Cat. No. HT501128, Thermo Fisher Scientific) for 24–48 hours at room temperature, embedded in paraffin, and stained with hematoxylin and eosin (HE). The slides of all tumors were reviewed to select appropriate blocks. For IHC, selected 4-5㎛-thick sections were cut and mounted on a glass slide, deparaf-finized and rehydrated. IHC was performed on a Roche Ventana Benchmark ULTRA automated stainer platform using the prediluted mouse anti-human β-hCG monoclonal antibody (Clone: OTI12H5, ZSGB-BIO, Cat. No. TA805383, Beijing, China) and the OptiView DAB Detection Kit (Cat. No. 760-700) according to the manufacturer's protocol. Appropriate positive control tissue (normal placental tissue) was used. Negative controls consisted of additional tumor sections, stained similarly but without the anti-β-hCG antibody.

**Table S1 Detailed information regarding the two methods used for the β-hCG assay.**

| Testing platform | Roche Cobas e 801 | Beckman DxI 800 |
| --- | --- | --- |
| Methodology | Electrochemiluminescence Immunoassay | Chemiluminescence Immunoassay |
| Assay kit | Elecsys HCG+β  Cat. No.: 07251025190 | Access Total βHCG (5th IS)  Cat. No.: A85264 |
| Reference range  for postmenopausal woman | ≤8.3 mIU/mL | 0.1-11.6 mIU/mL |
| Heterophile antibody blocking | Not specified | √ |
| On-board dilution system | Automatic dilution on Roche platform | UniCel DxI d‐CG5 |
| Calibrator | >10,000 mIU/mL | >1,350 mIU/mL |
| Hook effect-free upper limit of measurement | 750,000 mIU/mL | 1000,000 mIU/mL |
| Maximum tolerance of common interfering substance | Bilirubin: 66 mg/dL  Hemoglobin: 1000 mg/dL  Lipid emulsion: 2000 mg/dl  Biotin: 70 ng/mL  Rheumatoidfactor: 1200 IU/mL | Acetaminophen: 20 mg/dL  Acetylsalicylic acid: 65 mg/dL  Bilirubin: 40 mg/dL  Hemoglobin: 500 mg/dL  Heparin: 7200 U/dL  Human serum albumin: 6 g/dL  Ibuprofen: 50 mg/dL  Triglycerides: 3 g/dL  Multivitamins: 0.9%(v/v) |
| Maximum tolerance of Cross-reactant | LH: 4000 mIU/mL  FSH: 4000 mIU/mL  TSH: 2000 mIU/mL | LH: 103 mIU/mL  FSH: 1000 mIU/mL  TSH: 1 mIU/mL  ɑ-hCG: 500 mIU/mL |


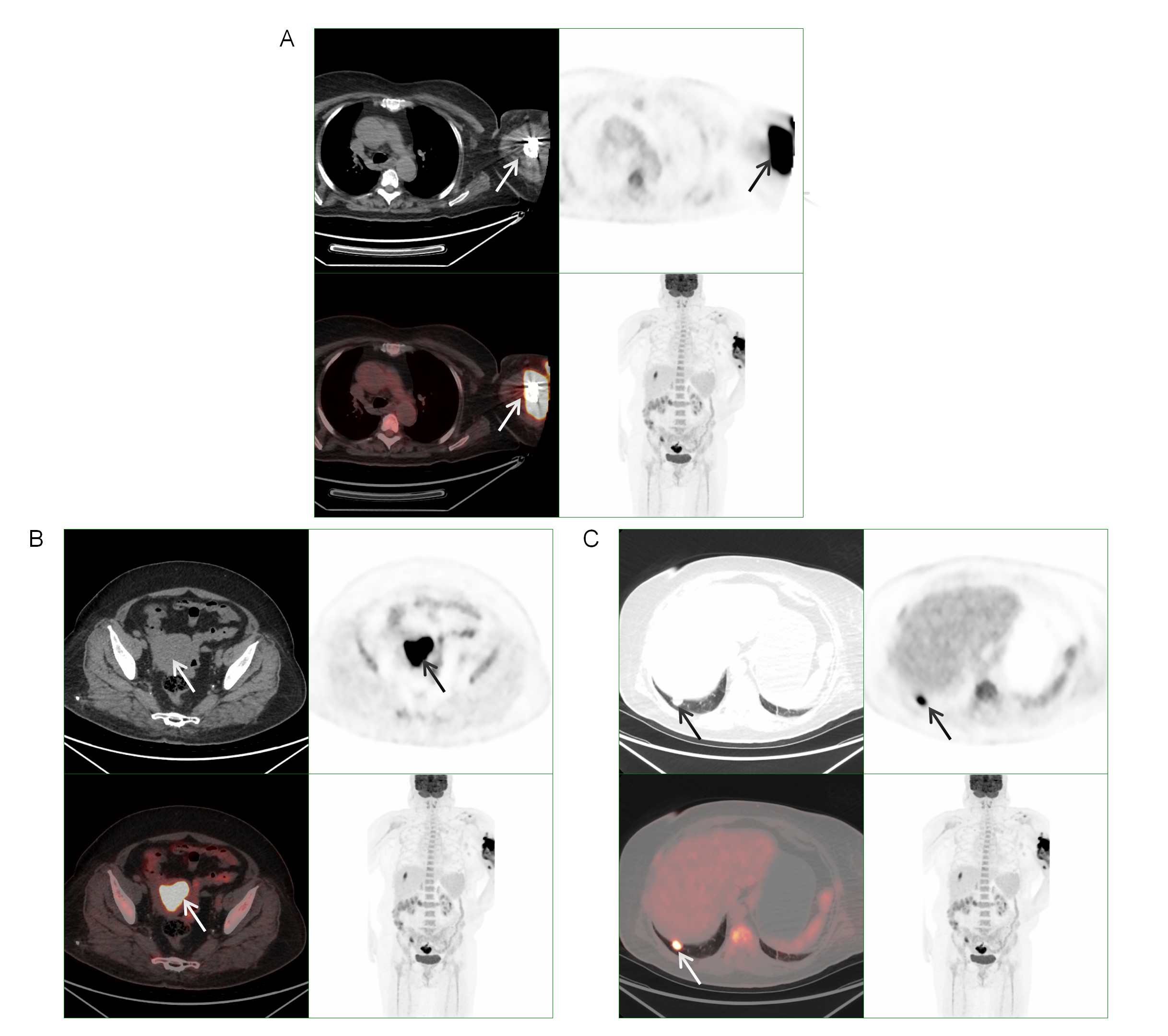


**Figure S1.** Representative diagnostic PET-CT images of multi-focal disease.

A: Imaging reveals ill-defined, hypermetabolic (SUVmax: 21.79) soft tissue masses around the humeral shaft, as well as a hypermetabolic (SUVmax: 6.87) focus in the humeral head. B: A hypermetabolic mass-like focus (2 x 1 cm, SUVmax: 14.03) within the uterine cavity, seen against a background of heterogeneous density. C: A poorly marginated, subpleural soft tissue nodule (1.5 cm in diameter) in the right lower lobe exhibited intense hypermetabolism (SUV max: 14.31).


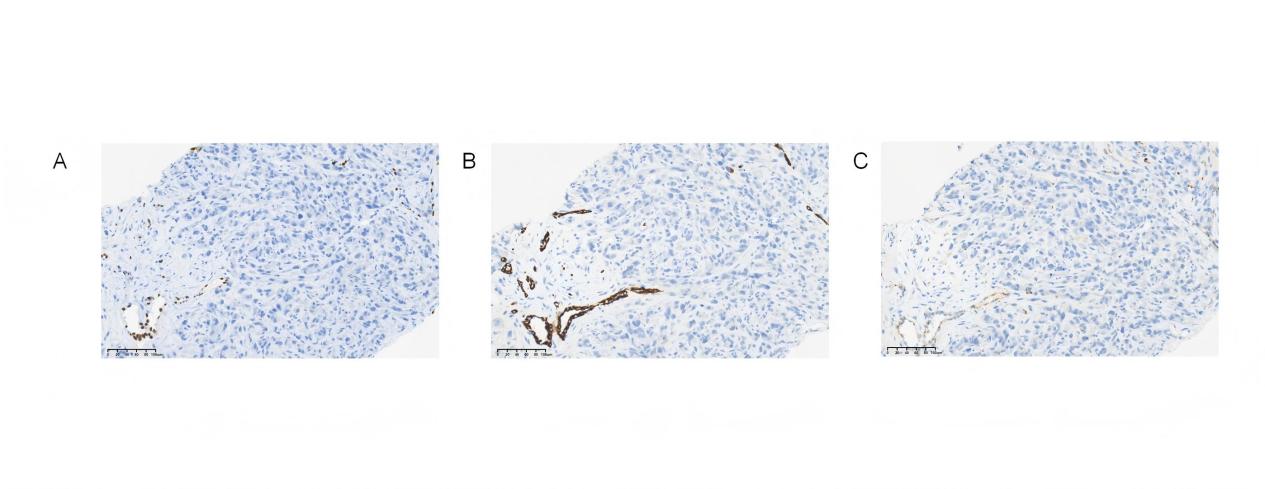


**Figure S2.** Immunohistochemical (IHC) findings at lung biopsy.

A: IHC staining of TTF-1. **B**: IHC staining of CK7. C: IHC staining of Napsin A. Scale bar: 100㎛.
