# Supplementary figures and images for: Paraneoplastic β-hCG secretion in a postmenopausal woman with sarcoma, endometrial carcinoma, and metastatic lung disease: a case report and review of the literature
Source: BMC Womens Health. 2026 Jan 26;26:115. doi: 10.1186/s12905-026-04298-1 (PMC12918461; doi:10.1186/s12905-026-04298-1)

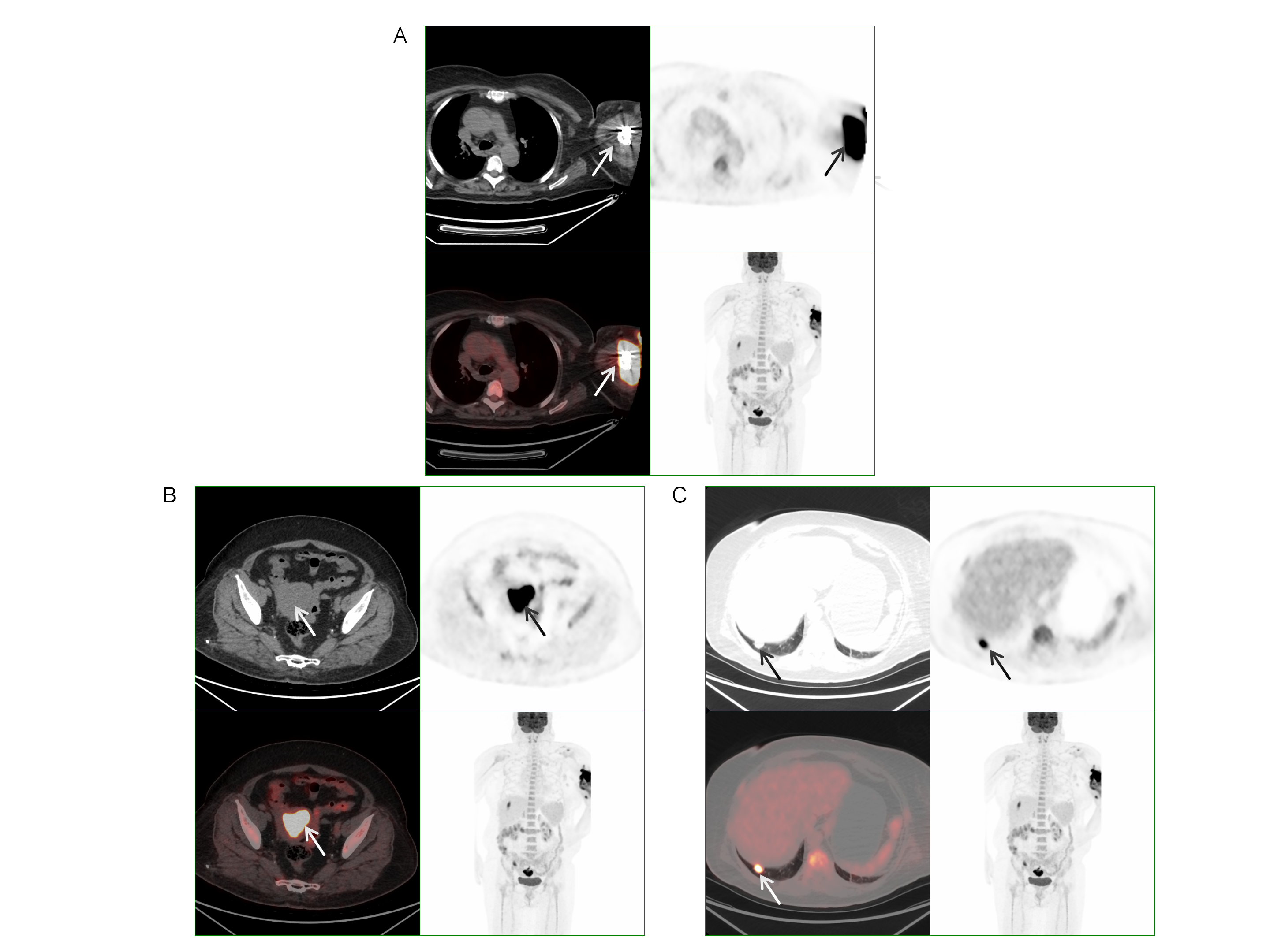

Supplement: Supplementary file 2 — Supplementary Material 2. [file 12905_2026_4298_MOESM2_ESM.tif]

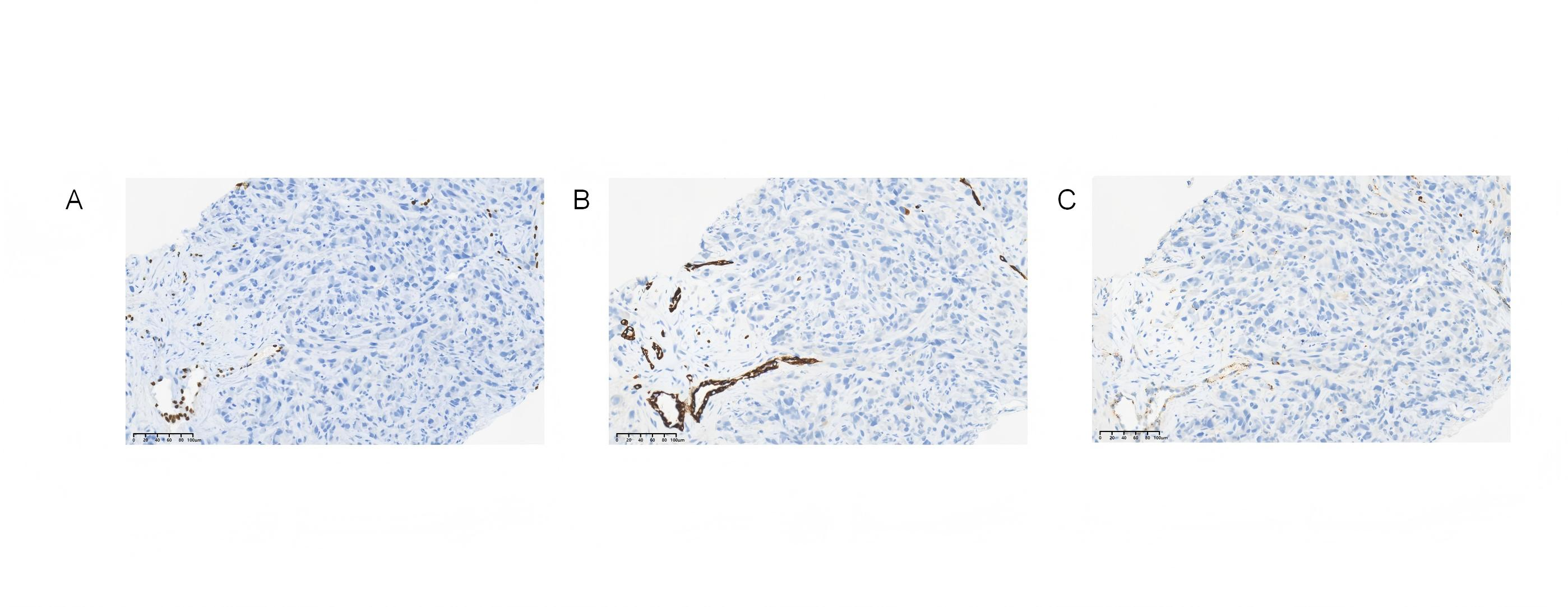

Supplement: Supplementary file 3 — Supplementary Material 3. [file 12905_2026_4298_MOESM3_ESM.tif]
